# Efficacy of prospective pharmacogenetic testing in the treatment of major depressive disorder: results of a randomized, double-blind clinical trial

**DOI:** 10.1186/s12888-017-1412-1

**Published:** 2017-07-14

**Authors:** Víctor Pérez, Ariana Salavert, Jordi Espadaler, Miquel Tuson, Jerónimo Saiz-Ruiz, Cristina Sáez-Navarro, Julio Bobes, Enrique Baca-García, Eduard Vieta, José M. Olivares, Roberto Rodriguez-Jimenez, José M. Villagrán, Josep Gascón, Josep Cañete-Crespillo, Montse Solé, Pilar A. Saiz, Ángela Ibáñez, Javier de Diego-Adeliño, AB-GEN Collaborative Group, Enric Álvarez, Fermín Mayoral-Cleries, Javier Quintero, Diego J. Palao, Luis Javier Irastorza, Rafael Navarro, María Luisa Barrigón, Marina Garriga, Lucía Villoria, Virginia Soria, José M. Rodao, Juan Castaño, Jordi Blanch, Cristobal Díez-Aja, Mercè Brat, José M. Mongil, Juan Miguel Garrido, Fernando Mora, Pedro M. Holgado, Roberto Sánchez-González, Alexandra Bagney, Eva Aguilar, María Paz García-Portilla, Gemma Safont, Joana Bauzà, Mercedes Martín-del Moral, Nazaret Cantero, Miquel Bernardo, Núria Rissech, Marta Puigmulé, Miquel Àngel Bonachera, José M. Menchón

**Affiliations:** 1 Centro de Investigación Biomédica en Red de Salud Mental (CIBERSAM), Av. Monforte de Lemos, 3-5, Madrid, Spain; 2 Institut de Neuropsiquiatria i Addiccions (INAD), Hospital del Mar, Institut Hospital del Mar d’Investigacions Mèdiques (IMIM), Departament de Psiquiatria, Universitat Autònoma de Barcelona, Barcelona, Spain; 3 AB-Biotics, S. A, Barcelona, Spain; 4 Departament of Psychiatry, Hospital Universitario Ramón y Cajal, Instituto Ramón y Cajal de Investigación Sanitaria (IRYCIS), Universidad de Alcalá, Madrid, Spain; 5 University Psychiatric Hospital, Institut Pere Mata, IISPV, Universitat Rovira Virgili, Reus, Spain; 6 Área de Psiquiatría, Facultad de Medicina, Universidad de Oviedo, Instituto Universitario de Neurociencias del Principado de Asturias (INEUROPA), Oviedo, Spain; 7 Departamento de Psiquiatría, Fundación Jiménez Díaz, IIS FJD, Madrid, Spain; 8 Hospital Universitario Rey Juan Carlos, Hospital Universitario Infanta Elena, Hospital General de Villalba, Universidad Autónoma de Madrid, Madrid, Spain; 9 Columbia University, New York, USA; 10 Department of Psychiatry and Psychology, Institute of Neuroscience, Hospital Clinic Barcelona, Institut d’Investigacions Biomèdiques August Pi i Sunyer (IDIBAPS), University of Barcelona, Barcelona, Spain; 11 Department of Psychiatry, Hospital Álvaro Cunqueiro, Complejo Hospitalario Universitario de Vigo, Instituto Biomédico Galicia Sur, Vigo, Spain; 12 Department of Psychiatry, Instituto de Investigación Hospital 12 de Octubre (i+12), Madrid, Spain; 13 Psychiatric Hospitalization Unit, Hospital General de Jerez de la Frontera, Jerez de la Frontera, Cádiz Spain; 14 Psychiatric Unit, Hospital Universitari Mútua Terrassa, Terrassa, Spain; 15 Mental Health Department, Hospital de Mataró, Consorci Sanitari del Maresme, Mataró, Spain; 16 Servei de Psiquiatria, Hospital de la Santa Creu i Sant Pau, Institut d’Investigació Biomèdica Sant Pau (IIB Sant Pau), Universitat Autònoma de Barcelona, Barcelona, Spain; 17 Department of Psychiatry, Hospital Universitari de Bellvitge, Institut d’Investigació Biomèdica de Bellvitge (IDIBELL), Carretera de la Feixa Llarga s/n, 08907 Hospitalet de Llobregat, Barcelona, Spain; 18 Departament de Ciències Clíniques, Facultat de Medicina, Universitat de Barcelona, Barcelona, Spain

**Keywords:** Depression, Pharmacogenetics, Precision medicine, Antidepressant response, Randomized clinical trial

## Abstract

**Background:**

A 12-week, double-blind, parallel, multi-center randomized controlled trial in 316 adult patients with major depressive disorder (MDD) was conducted to evaluate the effectiveness of pharmacogenetic (PGx) testing for drug therapy guidance.

**Methods:**

Patients with a CGI-S ≥ 4 and requiring antidepressant medication de novo or changes in their medication regime were recruited at 18 Spanish public hospitals, genotyped with a commercial PGx panel (Neuropharmagen®), and randomized to PGx-guided treatment (*n* = 155) or treatment as usual (TAU, control group, *n* = 161), using a computer-generated random list that locked or unlocked psychiatrist access to the results of the PGx panel depending on group allocation. The primary endpoint was the proportion of patients achieving a sustained response (Patient Global Impression of Improvement, PGI-*I* ≤ 2) within the 12-week follow-up. Patients and interviewers collecting the PGI-I ratings were blinded to group allocation. Between-group differences were evaluated using χ2-test or t-test, as per data type.

**Results:**

Two hundred eighty patients were available for analysis at the end of the 12-week follow-up (PGx *n* = 136, TAU *n* = 144). A difference in sustained response within the study period (primary outcome) was not observed (38.5% vs 34.4%, *p* = 0.4735; OR = 1.19 [95%CI 0.74-1.92]), but the PGx-guided treatment group had a higher responder rate compared to TAU at 12 weeks (47.8% vs 36.1%, *p* = 0.0476; OR = 1.62 [95%CI 1.00-2.61]), and this difference increased after removing subjects in the PGx-guided group when clinicians explicitly reported not to follow the test recommendations (51.3% vs 36.1%, *p* = 0.0135; OR = 1.86 [95%CI 1.13-3.05]). Effects were more consistent in patients with 1–3 failed drug trials. In subjects reporting side effects burden at baseline, odds of achieving a better tolerability (Frequency, Intensity and Burden of Side Effects Rating *Burden* subscore ≤2) were higher in the PGx-guided group than in controls at 6 weeks and maintained at 12 weeks (68.5% vs 51.4%, *p* = 0.0260; OR = 2.06 [95%CI 1.09-3.89]).

**Conclusions:**

PGx-guided treatment resulted in significant improvement of MDD patient’s response at 12 weeks, dependent on the number of previously failed medication trials, but not on sustained response during the study period. Burden of side effects was also significantly reduced.

**Trial registration:**

European Clinical Trials Database 2013-002228-18, registration date September 16, 2013; ClinicalTrials.gov NCT02529462, retrospectively registered: August 19, 2015.

**Electronic supplementary material:**

The online version of this article (doi:10.1186/s12888-017-1412-1) contains supplementary material, which is available to authorized users.

## Background

Major depressive disorder (MDD) is a leading cause of disability worldwide [1]. Actions to reduce the impact of depression on patients, families and healthcare systems are thus a public health priority. Despite the growing number of pharmacological treatments at our disposal, response and remission rates for antidepressants are not optimal [2] and psychiatry faces the challenge to improve utilization of current therapeutic tools. Besides failure with first- or second-line therapy, disability and economic costs associated with long-term depression are also linked to high rates of drug-induced adverse effects [3].

Common genetic variation has been estimated to explain up to 42% of variance in antidepressant response [4]; however, genome-wide association studies had been mostly unsuccessful in identifying individual risk variants and there are only a few examples on the use of pharmacogenetic information to guide treatment selection [5, 6]. The role of individual gene variants in the metabolism and response to psychotropic medications has been studied by several independent research groups and current evidence supports the contribution of certain genes to drug metabolism, safety or efficacy. Among them, the most replicated findings include cytochrome P450 genes (mainly *CYP2D6* and *CYP2C19*) and serotonin genes (*SLC6A4*, *HTR2C*, *HTR2A*) as well as the *ABCB1* transporter gene [7–10]. Recently, the Clinical Pharmacogenetics Implementation Consortium (CPIC) has published guidelines for drug selection and/or dosing of tricyclic antidepressants [11] and selective serotonin reuptake inhibitors [12] based on genotypes for *CYP2D6* and *CYP2C19*. Moreover, pharmacogenomics information has been incorporated into drug labels both in the US and EU [13, 14]. Pharmacogenetics-driven precision medicine based on prediction of therapeutic efficacy, tolerability and side-effects could help reduce the time spent until the ‘right drug at the right dose’ is attained [15]. However, for an adequate clinical implementation of pharmacogenetic testing in psychiatry, information from different genes influencing pharmacokinetic and pharmacodynamic aspects of drug response must be integrated, as well as practical tools are needed to facilitate the interpretation of the genotyping results and their translation into clinical practice [16]. Additionally, prospective randomized clinical trials (RCT) conducted in populations of adequate size are necessary to assess the clinical utility of pharmacogenetic testing in psychiatry. A recent systematic review reported that a limited number of studies have shown promise for the clinical utility of pharmacogenetic testing [17]. However, it was noted that the majority of studies were not randomized or blinded, indeed only two small single-center RCT [18, 19] have been conducted with a proper randomized double-blinded design. This review also pointed out that published studies did not assess at what point in the treatment of MDD pharmacogenetic testing should be utilized.

Neuropharmagen® (NFG®) is a pharmacogenomics-based precision medicine platform, developed by AB-Biotics SA (Barcelona, Spain), for managing psychiatric patients and to assist clinicians in drug selection and/or dosing choices. Previously, efficacy of pharmacogenetic testing with this test in the selection of psychiatric medication was retrospectively evaluated in 182 patients with various psychiatric diagnoses (depression, anxiety, schizophrenia and bipolar disorder, among others). This study showed that subjects whose treatment was prescribed following the test recommendations had 3.86-fold greater odds of improvement than patients whose treatment did not follow the test recommendations [20]. Also, stabilization rate at 3-month follow-up was significantly higher in individuals whose treatment followed the pharmacogenetic test results.

Here we present the results of a prospective, multicenter, randomized, double-blind clinical trial analyzing the clinical utility of pharmacogenetic testing with the Neuropharmagen platform. The aim of the study was to analyze the efficacy of pharmacogenetic information in the selection of drug treatments for MDD patients conducted under real-world clinical practice conditions. Clinical utility of the test is analyzed in terms of improvement in depression symptoms and in drug tolerability. Additionally, we examined the effect of the number of previous failed antidepressant trials on the utility of pharmacogenetic treatment guidance.

## Methods

The aim of this prospective, multicenter, randomized, double-blind, parallel controlled trial was to evaluate the effectiveness of pharmacogenetic (PGx) testing for drug therapy selection in major depressive disorder patients by comparing clinical outcomes in patients randomized to PGx-guided treatment or treatment as usual (control group). This randomized controlled trial adheres to the CONSORT guidelines [21].

### Study sample

Five hundred and twenty patients (both outpatients and inpatients) were enrolled from 18 hospitals and associated mental health centers in Spain from July 29, 2014 to June 15, 2015 (Additional file 1: Figure S1) and provided a saliva sample for DNA analysis. Eligible subjects were 18 years of age and over, with a principal diagnosis of major depressive disorder according to the Diagnostic and Statistical Manual of Mental Disorders (DSM-IV-TR) criteria. Inclusion criteria were: i) subjects with a clinician-rated score in the Clinical Global Impression-Severity (CGI-S) scale ≥4 both at screening and randomization visit [22]; ii) subjects who, according to the doctors’ assessment, required medication de novo or were receiving treatment and required substitution or addition of drug treatment with an antidepressant drug. To obtain a representative sample, individuals with secondary comorbid psychiatric and other medical illness could be included. Only primary psychiatric diagnoses other than MDD, pregnant and breastfeeding women, as well as patients requiring treatment with quinidine, cinacalcet and/or terbinafine (known CYP2D6 strong inhibitors) were excluded.

### Data collection

**Fig. 1:**
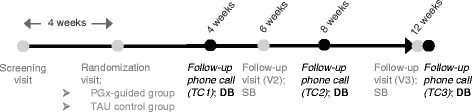
Schematic of the AB-GEN study procedures. TC: telephone controls; V: follow-up visits; DB: double-blind evaluation; SB: single-blind evaluation

Sociodemographic data, clinical diagnosis, duration of the depressive episode and current and past treatment for MDD as well as CGI-S scale (both clinician- and patient-rated) were recorded at the screening visit. Baseline (randomization visit) and follow-up visits at 6 and 12 weeks were conducted by study investigators on a single-blinded manner (Fig. 1), whereas blinded interviewers conducted telephone interviews at 4, 8 and 12 weeks to collect the Patient Global Impression of Improvement (PGI-I) scale (primary study variable [22] on a double-blinded manner. During the visits in person, drug treatments for MDD, changes and reasons for the changes and/or discontinuation were recorded. Clinical assessment performed by the treating psychiatrists (i.e. single-blinded) included the following scales: 17-item Hamilton Depression Rating Scale (HDRS-17) [23], Frequency, Intensity, and Burden of Side Effects Ratings (FIBSER) [24], clinician-rated and patient-rated CGI-S scale, Sheehan Disability Inventory (SDI) [25, 26], and Treatment Satisfaction with Medicines Questionnaire (SATMED-Q) [27].

### Genotyping and reporting of test results

At the screening visit, all study subjects provided a saliva sample for DNA extraction and genotyping of the genetic polymorphisms. DNA was extracted from patient saliva samples with the Genomic DNA Isolation Kit (Norgen Biotek Corp., Thorold, ON, Canada) following the manufacturer’s instructions. Genotyping of single nucleotide polymorphisms was performed by OpenArray® Technology on the QuantStudio™ 12 K Flex Real-Time PCR System (Thermo Fisher Scientific Inc., Waltham, MA USA) using a custom designed array (see Additional file 1: Table S1 for the list of polymorphisms analyzed). *CYP2D6* copy number analysis was performed in an Applied Biosystems® 7500 Real-Time PCR System using Hs04083572_cn and Hs04502391_cn TaqMan copy number assays targeting *CYP2D6* intron 2 and intron 6, respectively, and RNase P copy number assay as a reference (Thermo Fisher Scientific Inc., Waltham, MA USA).

The Neuropharmagen pharmacogenetic report (AB-Biotics SA, Barcelona, Spain) was accessible through a web-based computer-aided system (Additional file 1: Figure S2), provided information for 50 drugs (antidepressants, antipsychotics, mood stabilizers and other CNS drugs) and integrated three elements: (a) pharmacogenomic data derived from the analysis of genetic polymorphisms in 30 genes associated with drug efficacy, metabolism or specific adverse effects (Additional file 1: Table S1); (b) information on pharmacological interactions, involving psychotropic drugs as well as concomitant medications; and (c) data on specific clinical conditions and lifestyle influences. For each drug, the pharmacogenomics interpretative report (Additional file 1: Figure S3) highlights gene-drug interactions and provides drug-specific treatment recommendations as per FDA-approved drug labeling [13], published pharmacogenetic guidelines [11, 12, 28] and selected clinical studies [29–35]. For example, in a patient carrying the CYP2D6*4/*4 diplotype (predicted as a poor metabolizer phenotype), the antidepressant amitriptyline will be highlighted in yellow and the following recommendation will be associated to the drug: “Consider an alternative drug not metabolized by this pathway. If this drug is warranted, consider a 50% reduction of the recommended starting dose. Use therapeutic drug monitoring to guide dose adjustments”. Moreover, using a comprehensive built-in database of drug-drug, drug-clinical condition and drug-lifestyle factor interactions based on FDA-approved drug labeling, the web platform displays the most relevant alerts. The reporting algorithm prioritizes alerts using a color-coding system indicating with a red label alerts associated with adverse effects, with a yellow label alerts associated with drug metabolism variations, and with a green label alerts associated with increased likelihood of positive response. Whenever two or more alerts of different category are present for a particular drug, the summary table of the report highlights the most important alert whereas the detailed drug information section displays all the identified.

### Randomization, concealment and study groups

Subjects fulfilling all inclusion criteria and none of the exclusion criteria were included in the study and randomized to either the PGx-guided group or the control group (treatment as usual, TAU). Randomization was stratified by centre with a 1:1 ratio for intervention and control group, using a computer-generated random list. Patient blinding was ensured through a computer-assisted system: for each patient randomized to the PGx-guided group, the system provided the treating psychiatrists a numerical code to unlock their online access to the individual patient’s pharmacogenetics test report. At their discretion, treating psychiatrists could also check drug-drug, drug-clinical condition and drug-lifestyle factor interaction alerts in the reports online platform for patients in the PGx-guided group. For patients in the control group, clinicians did not have access to their reports until the end of the study and subjects were treated as usual without pharmacogenetic information. Patients in both study groups were treated by the same psychiatrists, who could choose whichever drug, drug combination and dosing schedule suited the best to each patient. For patients in the PGx-guided group, treating psychiatrists were asked to indicate whether, in their own judgment, the medication they prescribed was in accordance to the results provided by the test or not. As the treating psychiatrists were not blinded regarding patient allocation, assessment of the primary variable (PGI-I) was conducted by independent telephone interviewers who were blinded regarding patient allocation, thus ensuring a double-blind evaluation.

### Statistical analysis

The primary outcome of the study was the efficacy of the pharmacogenetic information in the selection of drug treatments for MDD considering the proportion of patients achieving a sustained response through a 12-week follow up period. A response was considered when the patient had a PGI-I score of 2 or less at a given phone interview (i.e. reported their condition was “Much better” or “Very much better”). A sustained response was achieved when a patient was classified as a responder on at least two consecutive evaluations, and maintained that status until the final visit of the study. The PGI-I scale was selected as the primary study variable as it allowed double-blinded evaluation of patient-rated improvement through phone interviews, and could be easily incorporated into interviews at 4, 8 and 12 weeks. Sample size was calculated to detect a significant difference with a sustained response around 30% in the control group and of 45% in the study group with alpha = 0.05 and power = 80%, resulting in a target N of 390 (195 per group) when assuming a 20% loss to follow-up. Adding an expected loss of 25% of patients between screening visit and randomization visit due to improvement of their condition or not requiring medication changes resulted in the total target N of 520. A per protocol population was defined by excluding subjects randomized to the PGx-guided group when their treating psychiatrists explicitly reported to have prescribed a treatment regime not in accordance to the test results. Secondary variables were response at the end of the 12-week follow-up (based on a PGI-I score of 2 or less), clinical progression (based on HDRS-17 score), severity of the disorder (based on the CGI-S score), tolerability of treatment (based on the FIBSER score), patient satisfaction with treatment (according to the SATMED-Q score), and patient disability (based on the SDI score). Regarding treatment tolerability, the cutoff for the acceptability of side effects was established at a FIBSER *Burden* subscore equal lower than 2, following the built-in recommendations in the FIBSER questionnaire. A descriptive statistics analysis for all variables was performed. χ2 tests or Student’s t-tests were used to compare pairwise differences between groups and between baseline and follow-up visits as per data type, while Pearson’s r was used to measure correlation between variables. Shapiro-Wilk test was used to verify normality of data. All reported *p*-values are raw *p*-values. Because 4 outcomes comparing the PGx-guided group to the control one were calculated on the primary variable (sustained response and response on week 12, full population and per-protocol population), a *p*-value threshold corrected for multiple comparisons was calculated. A key assumption of multiplicity correction methods such as Bonferroni or Sidak is the independence of the assessed outcomes. Thus, because of the existing correlation among the outcomes calculated on the primary variable, a correlation-corrected method was used [36], resulting in an adjusted *p*-value threshold of 0.0271 when considering the lowest correlation observed between these 4 variables (i.e. the more conservative correction). The effect of time on response rate within each study group was assessed using the Cochran-Armitage modification of the χ2 tests (to account for the order of visits). Effect sizes on the HDRS-17 scale as a function of the number of previously failed antidepressant therapies for the current episode were calculated using Hedge’s formula for Cohen’s *d* [37]*.* Post-hoc sub-analyses were performed in subjects having failed 1 to 3 previous medications. Statistical analyses were performed using SAS version 9.3 software (SAS Institute Inc., Cary, NC, USA).

## Results

Sociodemographic and clinical information at baseline is shown in Table 1 and patient flow through the study is summarized in Additional file 1: Figure S4. Of the 520 patients with a diagnosis of MDD enrolled in the study, 316 fulfilled inclusion criteria and were randomized (intent-to-treat population). Evaluated subjects had a mean age of 51.2 years (± 12.6), 63.6% were women and 92.4% Caucasian, and had a mean duration of MDD since diagnosis of the current depressive episode of 60.2 months (± 94.4), and a median of 14.1 months. At randomization, the mean HDRS-17 score was 19.2 (± 5.8). The main psychiatric comorbidities were anxiety disorder (35.8%) and substance abuse disorders (12.6%), and most patients were not drug-naïve (84.2%), with an average of 2.6 (± 2.2) previous antidepressant medication trials for the current episode, varying from 0 to 15. Similar scores were recorded at baseline in both the study and control groups in all questionnaires, except for the *Intensity* and *Burden* of side effects items of the FIBSER scale, which were significantly higher in the study group (Additional file 1: Table S2). The proportion of subjects not attending visits in the study and control groups was 7.1% vs 11.2%, respectively, while the proportion of subjects not answering telephone interviews for the primary variable was 12.3% vs 10.6%, respectively. Thus, there were no statistically significant differences in patient drop-outs (subjects not attending visits) nor in loss to follow-up for the primary variable (subjects not answering the telephone interviews).

**Table 1:** Demographic and clinical characteristics of the study population

		Study group	Control group	*p*-value
Subjects		155	161	
Age (years), mean (SD)		51.74 (12.05)	50.74 (13.12)	0.4801
Gender, n (%)	Female	99 (63.9)	102 (63.4)	0.9239
	Male	56 (36.1)	59 (36.6)	
Ethnicity (%)	Caucasian	145 (93.5)	147 (91.3)	0.6215
	Latin American	7 (4.5)	10 (6.2)	
	Other	3 (2.0)	4 (2.5)	
Time since diagnosis (months), mean (SD)		58.89 (93.29)	61.52 (95.80)	0.8050
Main diagnosis, n (%)	Major depression	146 (94.2)	148 (91.9)	0.7035
	Dysthymic disorder	5 (3.2)	8 (5.0)	
	Other non-specified depressive disorder	4 (2.6)	5 (3.1)	
Clinical Global Impression-Severity (CGI-S) scale, clinician-rated, mean (SD)		4.50 (0.62)	4.40 (0.57)	0.1663
Hamilton Depression Rating Scale (HDRS-17), mean (SD)		19.47 (5.96)	19.01 (5.71)	0.4818
Patient Global Impression of Improvement scale (PGI-I), single-blinded inclusion criterion, n (%)	No change	108 (69.7)	123 (76.4)	0.4026
	A little worse	26 (16.8)	20 (12.4)	
	Much worse	16 (10.3)	16 (9.9)	
	Very much worse	5 (3.2)	2 (1.2)	
Previous failed psychiatric medication trials, mean (SD)		2.55 (2.35)	2.57 (2.10)	0.9175

### Clinical efficacy on depression outcomes

**Fig. 2:**
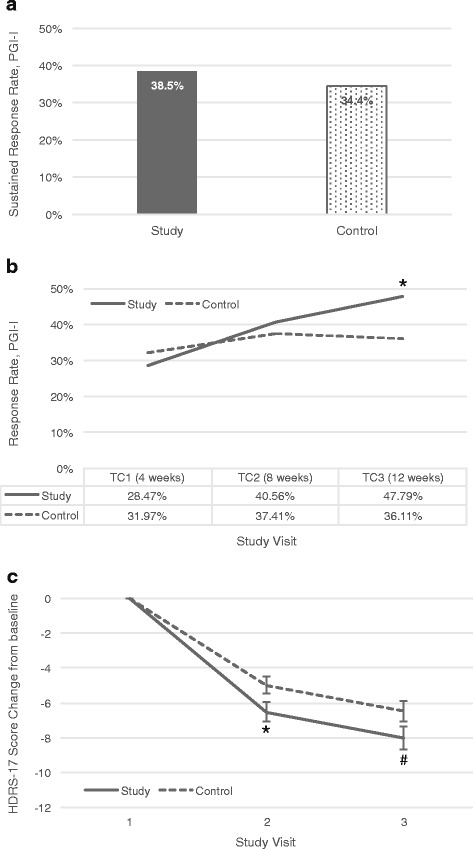
**a** Sustained response rate (PGI-I score ≤ 2 on two consecutive evaluations by phone interview). **b** Response rate based on subjects reporting a PGI-I score ≤ 2 and **c** change in HDRS-17 score in the total ITT population. TC: telephone controls; ^#^ *p* < 0.1; *p < 0.05

The PGI-I score was evaluated in a double-blinded manner at 4, 8 and 12 weeks. Differences in sustained response starting at 4 or 8 weeks could not be observed in this study (Fig. 2a). The number of responders at the end of the study (i.e. indicating their condition was “Much better” or “Very much better” on week 12) was nominally higher in the PGx-guided group than in the control group (47.8% vs 36.1%, *p* = 0.0476, OR = 1.62 [95%CI 1.00-2.61]). Moreover, in a within group analysis, the response rate in the PGx-guided group increased progressively in the three phone interviews from week 4 to week 12 (*p* = 0.0009), while the increase within the control group was not statistically significant (Fig. 2b).

In 17 of the subjects of the PGx-guided group, the treating psychiatrists reported to have prescribed medications in disagreement with the test results during the study period. Thus, the per-protocol analysis excluded these subjects from the PGx-guided group. Again, a significant difference in sustained response was not observed in this analysis, but the difference in the response rate at 12 weeks between the PGx-guided group and controls increased (51.3% vs 36.1%, *p* = 0.0135, OR = 1.86 [95%CI 1.13-3.05]). When correcting for multiplicity of analysis for the 4 comparisons of PGI-I between the PGx-guided and the control group (sustained response and response at 12 weeks, full population and per-protocol population), this *p*-value of 0.0135 is below the correlation-adjusted threshold calculated of 0.0271 (see Methods). Besides, response rate at 12 weeks among the 17 subjects excluded in the per-protocol analysis was very low (23.5%, *p* = 0.0323, compared to the remaining subjects of the PGx-guided group).

In the secondary analyses, improvement in depression rating scores was also statistically significant for the 17-item Hamilton Depression Rating Scale (HDRS-17) at 6 weeks (*p* = 0.0364) and a trend at 12 weeks (*p* = 0.0771) (Fig. 2c). Patients in the PGx-guided group presented a higher reduction in HDRS-17 (approximately a point and a half difference) than TAU control patients, that corresponded to Cohen’s *d* values of 0.25 and 0.21 at 6 and 12 weeks, respectively. Moreover, significant results favoring the PGx-guided treatment group were found in clinician-rated CGI-S, all three FIBSER indices and SATMED-Q total and partial scores, as well as the SDI Perceived Social Support partial score, but not in patients-rated CGI-S (Additional file 1: Table S3). Response and remission rates calculated post-hoc on the HDRS-17 (single-blinded) are reported in Additional file 1: Table S4, and baseline severity, as determined by HDRS-17, was found to impact the difference in response rate between PGx-guided and control groups. No significant differences were found in the distribution of antidepressants types prescribed in the PGx-guided and the control groups (Additional file 1: Table S5). Of note, PGI-I rating on week 12 was significantly correlated to change from baseline in both the clinician-rated CGI-S and HDRS-17 (*r* = −0.46 and *r* = −0.30, respectively, *p* < 0.0001 in both cases), indicating that the lower the PGI-I score (i.e. more improvement), the larger the reduction in CGI-S and HDRS scores. Also, PGI-I rating on week 12 was significantly correlated to sustained response (*r* = 0.56, *p* < 0.0001).

Because the PGx web platform also provides a tool to assess the impact of drug-drug, drug-clinical condition and drug-lifestyle factor interactions, the PGx sample was further split in two groups depending on whether the treating physicians had consulted the interactions tool. Responder rate was slightly higher in the group where interaction alerts had been consulted, compared to use of PGx alone (50.0% vs 45.8%), but the difference was not significant. Conversely, an opposite trend was observed in HDRS-17 score, as the reduction was slightly larger in the group using PGx information alone, both on week 6 and 12, but again the difference was not statistically significant.

**Fig. 3:**
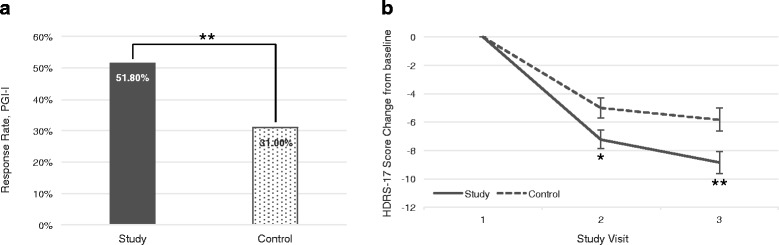
**a** Response rate based on subjects reporting a PGI-I score ≤ 2 and **b** change in HDRS-17 score in patients having received 1 to 3 previous treatments for the current depressive episode. *p < 0.05; **p < 0.01

Due to the large heterogeneity observed in the number of previous antidepressant medication trials for the current depressive episode, a sub-analysis was performed to evaluate the effect of this variable on the benefit of pharmacogenetic testing, by means of calculation of Cohen’s *d*. Study subjects with 1, 2 or 3 previous failed treatments for the current episode had a small clinical benefit compared to actively treated controls as seen by Cohen’s *d* calculated from the change in HDRS-17, whereas drug naïve subjects and those having received 4 or more medication trials did not (Additional file 1: Table S6). Among subjects having received 1 to 3 previous psychiatric treatments (*n* = 173), statistically significant differences were identified at 12 weeks in the percentage of patients with a positive response to treatment based on the PGI-I score (51.8% vs 31%, *p* = 0.0058, OR = 2.39 [95%CI 1.28-4.44]), and on the HDRS-17 score both at 6 weeks (*p* = 0.0237) and 12 weeks (*p* = 0.0083) (Fig. 3a and b). At 12 weeks, the mean and median reductions in HDRS-17 were 3 points larger in the study group than in the control group (Additional file 1: Table S7), resulting in a Cohen’s *d* value of 0.41.

### Medication tolerability

**Fig. 4:**
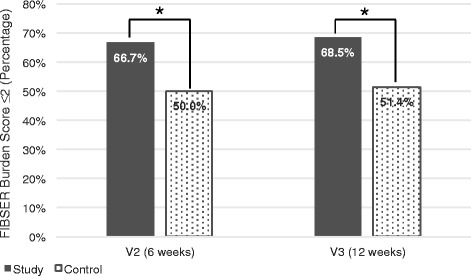
Differences in medication tolerability according to the FIBSER *Burden of side effects* subscore. The percentage of patients with scores ≤2 in the tolerability subpopulation are shown at 6 weeks (visit 2) and 12 weeks (visit 3) for the study and control groups. *p < 0.05

To assess the impact of pharmacogenetic testing on medication tolerability, subjects with a FIBSER *Burden* subscore of ≥1 at baseline were analyzed (*n* = 177). At baseline, this tolerability subpopulation did not display significant differences among groups in the FIBSER *Burden* domain score (Additional file 1: Table S8). Notably, the likelihood of reaching a FIBSER *Burden* score ≤ 2 (i.e. no general need to address side effects) was significantly higher in the PGx-guided treatment group after 6 weeks (66.7% vs 50.0%, *p* = 0.0294, OR = 2.00 [95%CI: 1.07 – 3.75]), and was maintained at 12 weeks (68.5% vs. 51.4%, *p* = 0.0260, OR = 2.06 [95%CI: 1.09 – 3.89]) (Fig. 4). To assess the independence between changes in depression symptoms and side effect burden, Pearson correlation was measured between the change in the FIBSER *Burden* subscore at 6 and 12 weeks and the change in HDRS-17 score on the same visits. Although the effect was statistically significant in both visits (*p* = 0.003 and *p* = 0.023), the effect sizes were very small (*r* = 0.18 and *r* = 0.14, respectively), suggesting that the improvement in medication tolerability made a significant but small effect on the improvement in the HDRS-17 scale.

## Discussion

To our knowledge this is the first large-scale multicenter, prospective, double-blind randomized clinical trial (RCT) to assess whether PGx-guided selection of treatment is more effective than unguided treatment in improving MDD patients’ response and poor drug tolerability. Our results show that achievement of sustained response within the 12-week follow-up period (primary outcome of the study) did not differ between the two groups. Positive results were found within 12 weeks of treatment based on the double-blind evaluation of patient-rated improvement (PGI-I), especially when the 17 subjects randomized to the PGx-guided group but being prescribed a drug regime not in accordance with the test results were excluded. Positive results were also observed within the first 6 weeks of treatment according to HDRS-17 change (single-blind).

Proper randomization and blinding are necessary because performing a genetic test has the potential to affect the attitude of the patient towards the post-test medication, resulting in increased adherence, as has been recently shown for other medical conditions [38]. In our study, we ensured double-blinding by using the patient rating of improvement (PGI-I scale), collected by blinded phone interviews, as the primary variable. The treating psychiatrists, which selected the drug regiment for patients either in the PGx-guided or TAU groups, could not be blinded and therefore phone interviews by independent raters were considered as the best option to guarantee double-blinded assessments. The present study has been conducted with conditions representative of real-world clinical practice. Most subjects enrolled were not drug-naïve (having received up to 15 previous courses of different psychiatric medication for the current depressive episode), and a substantial percentage of them displayed concurrent psychiatric disorders such as anxiety and substance abuse, while 17.8% displayed a depression of mild severity. In contrast, Phase III clinical trials of antidepressant efficacy commonly tend to employ more stringent inclusion/exclusion criteria, typically excluding subjects with comorbidities, those with HDRS-17 baseline severity of <19 and those with a current episode lasting more than 24 months [39]. In this regard, efficacy of antidepressant medication compared with placebo increases with severity of the disorder and tends to be smaller in patients with mild to moderate depression [39, 40]. In our study, comorbidities were not excluded and patients were selected according to a CGI-S score ≥ 4, which may explain the finding of a 17.8% of subjects with HDRS-17 score below 14 at randomization (baseline), the depression severity cutoff used in the STAR-D study [41]. Furthermore, our study included patients who had failed multiple previous treatments (65% of the population could be regarded as refractory).

A recent systematic review indicated that, up until now, only a handful of studies have examined the effects of combinatorial pharmacogenomics testing on clinical outcomes of adult MDD patients [17]. Of these, only two were randomized clinical trials, and none were multi-centric studies. In the first one, Winner and colleagues found a statistical trend for better outcomes in a trial conducted in 51 study subjects (26 pharmacogenetic-guided versus 25 unguided) with baseline HDRS-17 scores ≥14 [18]. Conversely, Singh [19] used a pharmacogenetic report to adjust drug dosages in 148 MDD patients (74 guided versus 74 unguided) and showed patients in the guided group had a 2.52-fold increased likelihood of remission. Of note, the latter included subjects with HDRS-17 scores over 18 only, and excluded smokers, patients with psychiatric comorbidities, and those receiving known inducers/inhibitors of CYP2C19, CYP2D6 and ABCB1. Although with promising results, both studies were conducted in populations of patients of modest size and thus additional data was needed to firmly establish the utility of pharmacogenetic testing for the treatment of MDD.

Therefore, regardless of the high heterogeneity of the AB-GEN full study population in terms of depression severity, length of diagnosis and comorbidities, statistically significant differences were observed in the PGx-guided treatment group versus the unguided group, although effect sizes were modest. The definition of sustained response in this study required at least obtaining significant differences already at 8 weeks, which may be too short a follow-up period, especially in a design seeking to demonstrate superiority against treatment as usual (opposed to trials intending to prove non-inferiority). The statistically significant increase in response rate in the PGx-guided group during the study period and the difference observed against the control group at 12 weeks raise the question of whether a longer follow-up time could confirm a difference in sustained response.

The effects of PGx-guided treatment on response (PGI-I) and HDRS-17 change were more consistent and clinically relevant in subjects with 1 to 3 unsuccessful previous drug trials, where a 2.39-fold increase in the odds of response was found. Hence, our results suggest that use of pharmacogenetic information to guide treatment adjustments would be justified if the traditional first line of treatment fails. Our results are in agreement with previous studies reporting that pharmacogenetic tools are effective in patients that failed a previous medication trial [18, 19, 42]. Moreover, the results of this study are consistent with previous data from a retrospective naturalistic study also conducted with Neuropharmagen [20]. However, our results also indicate that pharmacogenetics tools may be of little benefit to those patients with large numbers of unsuccessful medication attempts. We hypothesize this could be due to those patients having less untested therapeutic alternatives remaining, and thus the reduction in uncertainty caused by the test would be smaller. Future studies should attempt to assess this hypothesis.

Remarkably, the present study is also the first prospective multicenter double-blind RCT indicating that the use of a pharmacogenetic test report improves medication tolerability as suggested by a statistically significant reduction in the burden of side-effects. Non-adherence is a global challenge for psychiatry and has been linked to poorer outcomes, while improved tolerability facilitates long-term adherence. In the present study, a very small correlation was found between the change in HDRS-17 and the change in side effect burden on the same visits, suggesting that the improved tolerability is not the cause of the improvement in depression severity during the 12-week follow-up period.

The pharmacogenetic tool used in this study also allows physicians to check for potential drug-drug, drug-medical condition and drug-lifestyle factor interactions. Therefore, we compared those patients whose treating psychiatrists had consulted for interaction alerts to those whose treating psychiatrist had not, within the PGx group. A slight increase in response rate was observed in the former group, yet the opposite trend was observed regarding the reduction in HDRS-17, the differences being not significant in either case. Also, it must be noted that the number of subjects displaying real interactions was not determined, and could differ between the two subgroups, thus compromising their comparability. Therefore, no conclusions could be drawn regarding whether the addition of interaction alerts to pharmacogenetic information had a significant impact.

Personalization of psychiatric treatments using pharmacogenetic information is emerging as a valuable tool to identify in advance which medications will be more effective, which will require dose adjustments or which may cause meaningful adverse reactions. Besides a clinically-demonstrated effect on efficacy and tolerability, for pharmacogenetic data to represent a real benefit for psychiatric patients it needs to be seamlessly integrated into clinical practice. Drug response and tolerability profiles depend on the combined effects of different genes as well as environmental and clinical factors. In this regard, the translation of the combined effect of different genes into actionable recommendations has been previously shown to outperform the effect of single genes [43]. The Neuropharmagen web-based platform uses a proprietary combinatorial approach to translate pharmacogenetic as well as pharmacological information into clinical actionable recommendations. In the case of drug-metabolizing enzymes polymorphisms, international guidelines (Clinical Pharmacogenetics Implementation Consortium genotype-driven dose adjustments, FDA-approved drug labelling) are included in the recommendations when available.

A number of barriers have been noted for the widespread adoption of precision medicine, such as insufficient evidence generation, data sharing and slow uptake of genomic information into clinical care [44]. In this regard, a multi-centric, double-blinded RCT design such as the one used in our study represents the gold standard for evidence generation, while having performed the study in a naturalistic scenario allows to account for the limitations in sharing and uptaking of pharmacogenetic data in a real-life scenario.

## Conclusions

The AB-GEN study contributes to demonstrate that the use of a pharmacogenetic-based precision medicine platform may have a significant impact on clinical improvement of MDD patients and reduction of drug side effects compared to standard of care, even if results in sustained response rates before the end of the 12-week follow-up were similar in the studied groups. The effect was especially relevant in subjects with one to three previous failed antidepressant trials, but not in drug-naïve patients or those with more than 3 failed drug trials. This study does have several limitations. Firstly, personnel of AB-Biotics (developer of Neuropharmagen®) collaborated in the analysis of the results, the interpretation of the study and the preparation of the manuscript. Secondly, the clear majority of participating patients were of Caucasian origin. Thus, application of findings to other ethnic groups should be considered with care. Additionally, we acknowledge the limitations of using a simple scale, such as PGI-I, as the primary outcome assessment. Moreover, epigenetic factors are not included in current pharmacogenomic testing algorithms and thus were not considered in this study. Future approaches analyzing genetic as well as epigenetic factors may increase the predictive capability of such tools. Also, because most subjects were already on treatment at study entry and some were under polytherapy, we could not clearly establish whether medication changes occurring during the study affected drugs prescribed in the randomization visit, drugs prescribed before the randomization visit and kept at the same dose, or prescribed before the randomization visit but whose dosing had been changed according to the tests results. However, PGx testing may report several therapeutic alternatives or complementary drug options for a given patient, and thus changes in medication after randomization visit in a naturalistic setting cannot be directly related to a success or failure of a PGx test to deliver useful information. This further highlights the importance of large, randomized, multi-centric studies to assess the overall practical effect of using vs not using PGx information. Finally, additional studies will be needed for independent replication as well as to confirm these findings in other psychiatry diagnoses such as bipolar disorder or schizophrenia.

In summary, the present study found that PGx-guided prescription has potential to help improve both efficacy and tolerability compared to treatment as usual in a naturalistic setting, but such clinical utility likely depends on the patient profile, and should be replicated in studies with different ethnicities.

## Additional files

Additional file 1: Supplementary information. **Figure S1–4** and **Tables S1–8**. (DOCX 695 kb)

Additional file 2: List of Ethic Committees that approved the AB-GEN trial. Full names of the all ethics committees that approved the trial at each participating hospital. (DOCX 13 kb)

## Abbreviations

CGI-S: Clinical Global Impression-Severity
CNS: Central Nervous System
CPIC: Clinical Pharmacogenetics Implementation Consortium
DSM-IV-TR: Diagnostic and Statistical Manual of Mental Disorders, 4th Edition, Text Revision
FIBSER: Frequency, Intensity, and Burden of Side Effects Ratings
HDRS-17: 17-item Hamilton Depression Rating Scale
IRB: Institutional Review Board
MDD: Major depressive disorder
PGI-I: Patient Global Impression of Improvement Scale
PGx: Pharmacogenetics
RCT: Randomized clinical trials
SATMED-Q: Treatment Satisfaction with Medicines Questionnaire
SDI: Sheehan Disability Inventory
TAU: Treatment as usual
