# Supplementary material for: Efficacy of prospective pharmacogenetic testing in the treatment of major depressive disorder: results of a randomized, double-blind clinical trial
Source: BMC Psychiatry. 2017 Jul 14;17:250. doi: 10.1186/s12888-017-1412-1 (PMC5513031; doi:10.1186/s12888-017-1412-1)
Supplement: Supplementary file 1 — Supplementary information. Figure S1–4 and Tables S1–8. (DOCX 695 kb) [file 12888_2017_1412_MOESM1_ESM.docx]

**Additional file**

**Figure S1**. Participating centers and patients included (study and control groups) and treating psychiatrists in each center.


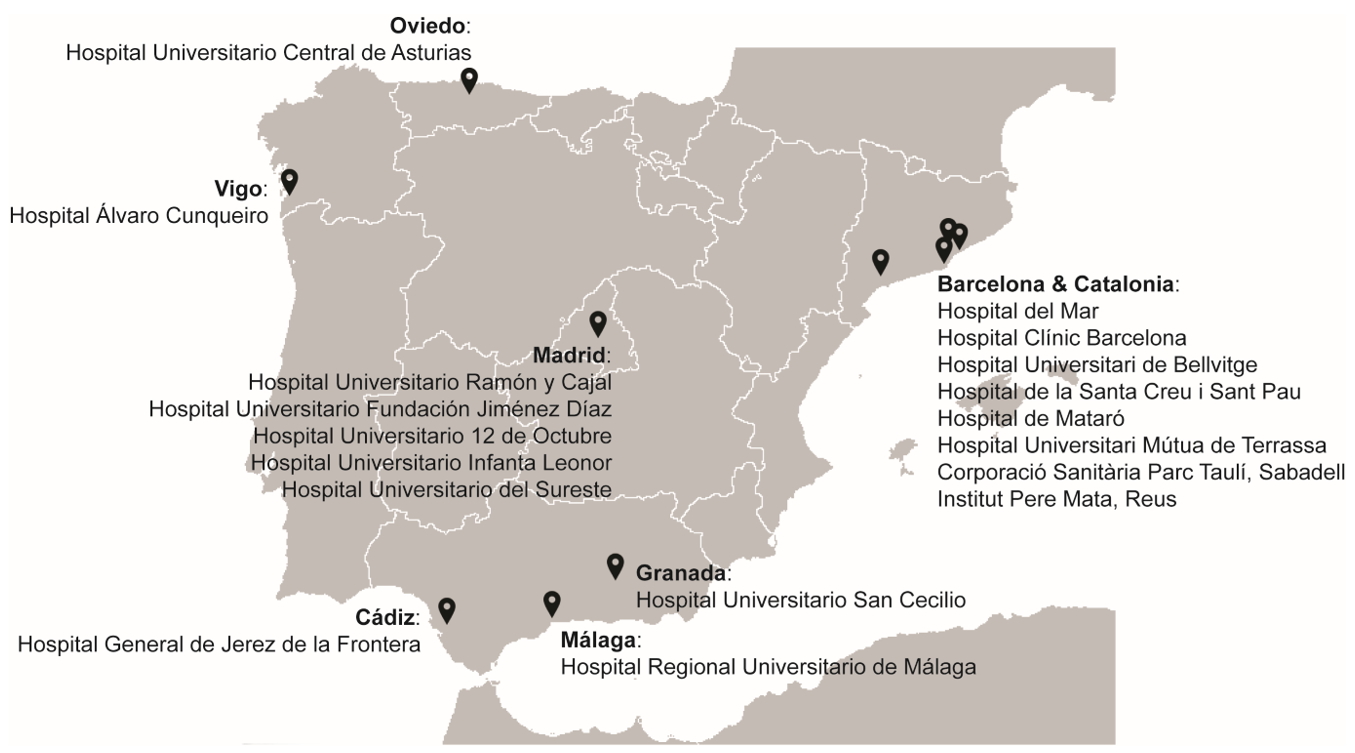


| **Participating Center** | **Psychiatrists** | **Patients (Study group)** | **Patients (Control group)** |
| --- | --- | --- | --- |
| Institut Pere Mata, Reus | 3 | 18 | 20 |
| Hospital Universitario Ramón y Cajal, Madrid | 3 | 18 | 18 |
| Hospital Universitario Central de Asturias, Oviedo | 3 | 16 | 17 |
| Hospital del Mar, Barcelona | 11 | 17 | 15 |
| Hospital Universitario F. Jiménez Díaz, Madrid | 3 | 11 | 13 |
| Hospital Clínic, Barcelona | 5 | 11 | 11 |
| Hospital Álvaro Cunqueiro, Vigo | 4 | 9 | 8 |
| Hospital Universitario 12 de Octubre, Madrid | 11 | 7 | 9 |
| Hospital Universitari de Bellvitge, Barcelona | 2 | 7 | 8 |
| Hospital General de Jerez de la Frontera, Cádiz | 7 | 7 | 7 |
| Hospital de la Santa Creu i Sant Pau, Barcelona | 6 | 6 | 7 |
| C. S. Maresme (Hospital de Mataró) | 1 | 5 | 6 |
| Hospital Universitari Mútua Terrassa, Terrassa | 6 | 5 | 5 |
| Hospital Universitario Infanta Leonor, Madrid | 3 | 4 | 5 |
| Hospital Regional Universitario de Málaga | 5 | 5 | 4 |
| Corporació Sanitària Parc Taulí, Sabadell | 5 | 4 | 3 |
| Hospital Universitario del Sureste, Madrid | 3 | 3 | 3 |
| Complejo Hospitalario Universitario de Granada | 3 | 2 | 2 |

**Figure S2**. Schematic of the Neuropharmagen/NeuroHealthDx personalized medicine technology platform for managing psychiatric patients. A web-based computer-aided system integrates three elements: (a) patient’s pharmacogenomic data; (b) pharmacological interactions; (c) environmental interactions.

Algorithm


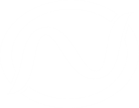


Genotype data

Genetic Analysis Platform

Gene-drug interactions

**Report**

Drug-specific recommendations

**PHARMACOLOGY**

**PHARMACOGENOMICS**

**ENVIRONMENTAL INFLUENCES**

Drug-specific recommendations

Clinical condition-drug interactions

Drug-drug interactions

Database

Drug labeling

PGx Clinical guidelines

PGx

Report

Other PGx info

**Figure S3.** Example of a Neuropharmagen pharmacogenomics interpretative report for one de-identified study subject, showing (a) the color coding classification of drugs and (b) detailed information for one of the drugs.

**
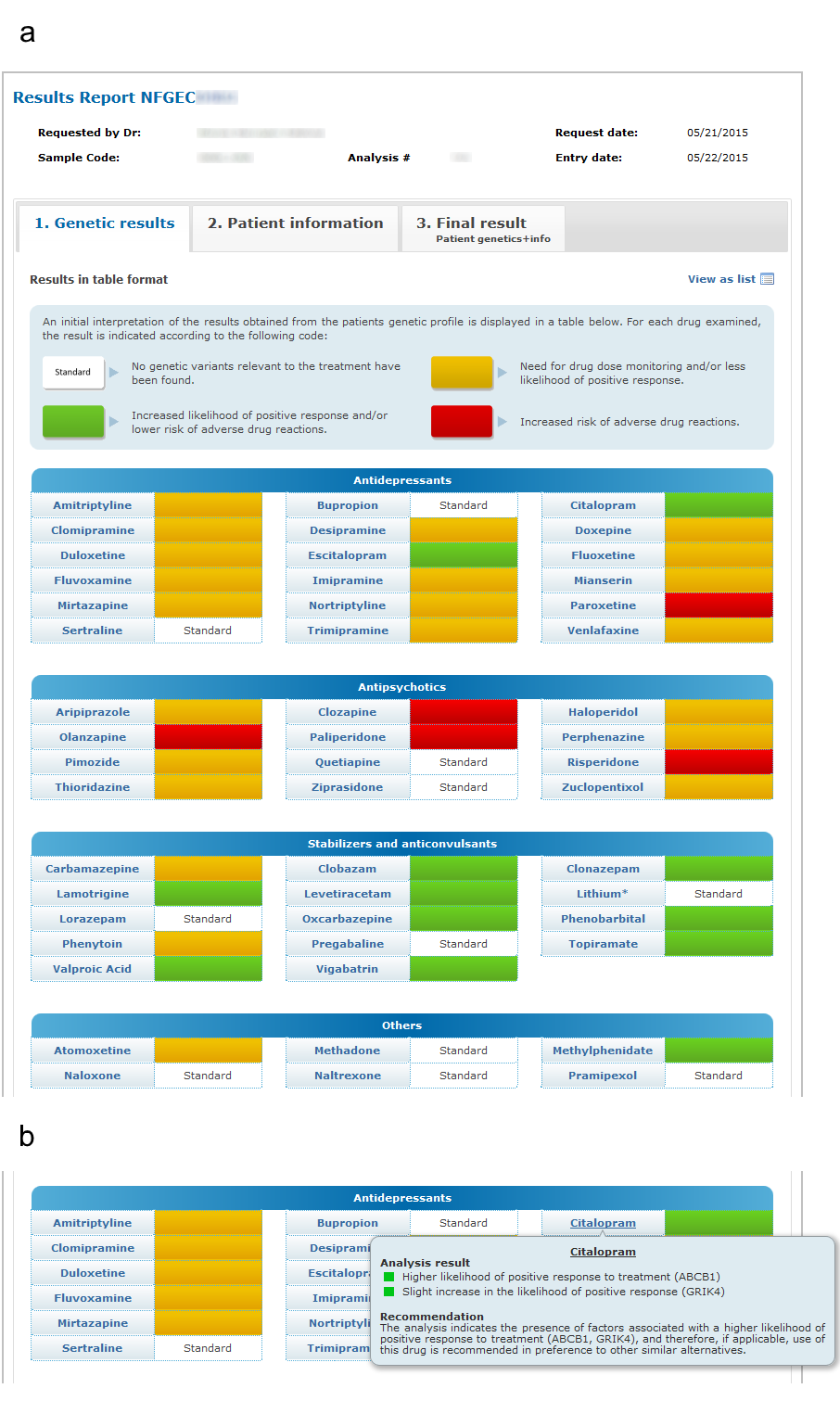
**

**Figure S4**. CONSORT flow diagram

Not meeting one or more inclusion criteria for randomization (n=204)

- Not fulfilling CGI-S/PGI-I cut-off (n=99)
- Not requiring drug changes at randomization (n=105)

Assessed for eligibility (n=520)

PGI-I (n=144)

PGI-I (n=136)

## **12-week phone call**

## **12-week follow-up visit**

## **8-week phone call**

PGI-I (n=143)

PGI-I (n=147)

HDRS-17 (n=143)

FIBSER (n=143)

CGI-S, clinician-rated (n=144)

SATMED-Q (n=143)

SDI (n=143)

(did not attend visit: n=11)

HDRS-17 (n=142)

FIBSER (n=143)

CGI-S, clinician-rated (n=143)

SATMED-Q (n=143)

SDI (n=143)

(did not attend visit: n=18)

HDRS-17 (n=146)

FIBSER (n=148)

CGI-S, clinician-rated (n=148)

SATMED-Q (n=148)

SDI (n=148)

(did not attend visit: n=13)

HDRS-17 (n=146)

FIBSER (n=147)

CGI-S, clinician-rated (n=147)

SATMED-Q (n=144)

SDI (n=146)

(did not attend visit: n=8)

## **6-week follow-up visit**

PGI-I (n=144)

PGI-I (n=147)

## **4-week phone call**

Study group

(PGx-guided treatment; n= 155)

Control group

(Treatment as usual; n=161)

Randomized (n=316)

**Table S1**. List of genes and polymorphisms analyzed

| **Gene symbol** | **Gene name** | **Polymorphisms** |
| --- | --- | --- |
| *ABCB1* | ATP binding cassette subfamily B member 1 | rs2235048, rs11983225 |
| *AKT1* | V-akt murine thymoma viral oncogene homolog 1 | rs1130214 |
| *BDNF* | Brain-derived neurotrophic factor | rs6265 |
| *CACNG2* | Calcium channel, voltage-dependent, gamma subunit 2 | rs2284017 |
| *CES1* | Carboxylesterase 1 | rs71647871 |
| *COMT* | Catechol-O-methyltransferase | rs4680 |
| *CRHR1* | Corticotropin releasing hormone receptor 1 | rs4792888 |
| *CYP1A2* | Cytochrome P450 family 1 subfamily A member 2 | *1, *1F |
| *CYP2B6* | Cytochrome P450 family 2 subfamily B member 6 | *1, *6 |
| *CYP2C19* | Cytochrome P450 family 2 subfamily C member 19 | *1, *2, *3, *5, *7, *8, *17, *27 |
| *CYP2C9* | Cytochrome P450 family 2 subfamily C member 9 | *1, *2, *3, *6, *8, *27 |
| *CYP2D6* | Cytochrome P450 family 2 subfamily D member 6 | *1, *2, *3, *4, *5, *6, *7, *8, *9, *10, *11, *12, *14, *15, *17, *19, *20, *29, *30, *35, *40, *41, *69, *1xN, *2xN, *35X2 |
| *CYP3A4* | Cytochrome P450 family 3 subfamily A member 4 | *1, *22 |
| *DDIT4* | DNA damage inducible transcript 4 | rs1053639 |
| *DRD3* | Dopamine receptor D3 | rs963468 |
| *EPHX1* | Epoxide hydrolase 1, microsomal (xenobiotic) | rs1051740 |
| *FCHSD1* | FCH and double SH3 domains 1 | rs456998 |
| *GRIK2* | glutamate receptor, ionotropic, kainate 2 | rs2518224 |
| *GRIK4* | glutamate receptor, ionotropic kainate 4 | rs1954787 |
| *HLA-A* | Major histocompatibility complex, class I, A | rs1061235 |
| *HTR1A* | 5-HT (serotonin) receptor 1A, G protein-coupled | rs10042486 |
| *HTR2A* | 5-HT (serotonin) receptor 2A, G protein-coupled | rs6311, rs6314, rs9316233 |
| *HTR2C* | 5-HT (serotonin) receptor 2C, G protein-coupled | rs1414334 |
| *LPHN3* | Latrophilin 3 | rs6551665 |
| *NEFM* | Neurofilament, medium polypeptide | rs1379357, rs1457266 |
| *OPRM1* | Opioid receptor, mu 1 | rs1799971 |
| *RGS4* | Regulator of G-protein signaling 4 | rs2661319 |
| *RPTOR* | Regulatory associated protein of MTOR, complex 1 | rs7211818 |
| *SLC6A4* | Solute carrier family 6 (neurotransmitter transporter), member 4 | 5-HTTLPR |
| *UGT2B15* | UDP glucuronosyltransferase 2 family, polypeptide B15 | rs1902023 |

**Table S2**. Results for baseline visit, ITT population

| **Results for baseline visit, ITT population** | | | | | |
| --- | --- | --- | --- | --- | --- |
| **End point** |  | **Total** | **Study Group** | **Control Group** | **p-value (1)** |
| CGI-S by patient (Visit 1) | Average (SD) | 4.74 (0.79) | 4.75 (0.81) | 4.73 (0.78) | 0.7535 |
| FIBSER - Frequency  (Visit 1) | Average (SD) | 2.10 (2.23) | 2.32 (2.23) | 1.88 (2.21) | 0.0791 |
| FIBSER - Intensity  (Visit 1) | Average (SD) | 1.94 (1.88) | 2.18 (1.86) | 1.71 (1.87) | **0.0272** |
| FIBSER - Burden  (Visit 1) | Average (SD) | 1.74 (1.76) | 1.99 (1.83) | 1.50 (1.66) | **0.0157** |
| SDI - Disability  (Visit 1) | Average (SD) | 22.53 (6.04) | 22.10 (6.34) | 22.94 (5.71) | 0.2223 |
| SDI - Stress  (Visit 1) | Average (SD) | 7.46 (2.27) | 7.42 (2.27) | 7.50 (2.27) | 0.7709 |
| SDI - Social support perceived  (Visit 1) | Average (SD) | 5.79 (2.92) | 5.55 (2.91) | 6.03 (2.91) | 0.1449 |
| SATMED-Q  (Visit 1) | Average (SD) | 54.16 (15.82) | 53.44 (16.07) | 54.86 (15.59) | 0.4333 |
| SATMED - Side effects (Visit 1) | Average (SD) | 67.72 (35.43) | 66.34 (35.13) | 69.06 (35.77) | 0.5015 |
| SATMED - Treatment effectiveness  (Visit 1) | Average (SD) | 34.04 (24.39) | 33.39 (25.27) | 34.67 (23.58) | 0.6464 |
| SATMED - Convenience of use (Visit 1) | Average (SD) | 68.81 (26.36) | 68.38 (26.74) | 69.23 (26.07) | 0.7773 |
| SATMED - Impact on activities of daily living (Visit 1) | Average (SD) | 31.68 (26.03) | 31.73 (26.08) | 31.63 (26.06) | 0.9737 |
| SATMED - Medical care (Visit 1) | Average (SD) | 74.07 (25.05) | 75.41 (23.69) | 72.77 (26.31) | 0.3555 |
| SATMED - General satisfaction  (Visit 1) | Average (SD) | 55.10 (27.36) | 52.70 (27.31) | 57.43 (27.30) | 0.1309 |
| (1) Chi-Square or ANOVA test, as per data type | | | | | |

**Table S3**. Efficacy results, ITT population

| **Efficacy results, ITT population** | | | | | |
| --- | --- | --- | --- | --- | --- |
| **Variable** |  | **Total** | **Study Group** | **Control Group** | **p-value (1)** |
| CGI-S by investigator | Visit 1 to 2 | -0.60 (0.86) | -0.67 (0.85) | -0.53 (0.86) | 0.1433 |
|  | Visit 1 to 3 | -1.00 (1.14) | -1.14 (1.13) | -0.87 (1.13) | **0.0425** |
|  | ≤ 3 at Visit 2 | 96 (32.5%) | 50 (34.0%) | 46 (31.1%) | 0.5909 |
|  | ≤ 3 at Visit 3 | 130 (45.3%) | 75 (52.1%) | 55 (38.5%) | **0.0205** |
| CGI-S by patient | Visit 1 to 2 | -0.71 (1.12) | -0.77 (1.09) | -0.65 (1.16) | 0.3595 |
|  | Visit 1 to 3 | -0.98 (1.38) | -1.09 (1.37) | -0.87 (1.38) | 0.1844 |
|  | ≤ 3 at Visit 2 | 85 (28.8%) | 43 (29.3%) | 42 (28.4%) | 0.8685 |
|  | ≤ 3 at Visit 3 | 108 (37.6%) | 58 (40.3%) | 50 (35.0%) | 0.3529 |
| FIBSER - Frequency of side effects | Visit 1 to 2 | -0.20 (2.60) | -0.52 (2.55) | 0.13 (2.62) | **0.0316** |
|  | Visit 1 to 3 | -0.47 (2.37) | -0.68 (2.35) | -0.25 (2.38) | 0.1280 |
| FIBSER - Intensity of side effects | Visit 1 to 2 | -0.14 (2.17) | -0.43 (2.12) | 0.14 (2.19) | **0.0244** |
|  | Visit 1 to 3 | -0.34 (1.98) | -0.60 (2.01) | -0.09 (1.92) | **0.0303** |
| FIBSER - Burden of side effects | Visit 1 to 2 | -0.16 (2.00) | -0.46 (2.03) | 0.14 (1.93) | **0.0105** |
|  | Visit 1 to 3 | -0.30 (1.88) | -0.57 (2.00) | -0.01 (1.72) | **0.0125** |
| SDI - Disability | Visit 1 to 2 | -2.99 (7.35) | -2.91 (7.04) | -3.06 (7.66) | 0.8606 |
|  | Visit 1 to 3 | -4.31 (8.10) | -4.66 (8.32) | -3.96 (7.89) | 0.4721 |
| SDI - Stress | Visit 1 to 2 | -1.27 (2.68) | -1.26 (2.63) | -1.27 (2.73) | 0.9698 |
|  | Visit 1 to 3 | -1.47 (3.09) | -1.67 (3.30) | -1.28 (2.86) | 0.2862 |
| SDI - Social support perceived | Visit 1 to 2 | 0.22 (2.60) | 0.41 (2.43) | 0.02 (2.75) | 0.1979 |
|  | Visit 1 to 3 | 0.41 (2.96) | 0.76 (2.96) | 0.06 (2.94) | **0.0479** |
| SATMED-Q | Visit 1 to 2 | 7.23 (18.86) | 10.06 (18.66) | 4.45 (18.69) | **0.0117** |
|  | Visit 1 to 3 | 10.90 (20.58) | 13.01 (21.34) | 8.76 (19.63) | 0.0844 |
| SATMED - Side effects | Visit 1 to 2 | 5.27 (37.39) | 7.75 (35.68) | 2.82 (38.98) | 0.2636 |
|  | Visit 1 to 3 | 6.79 (38.17) | 8.33 (38.60) | 5.24 (37.80) | 0.4977 |
| SATMED - Treatment effectiveness | Visit 1 to 2 | 10.28 (31.58) | 14.22 (30.43) | 6.37 (32.31) | **0.0349** |
|  | Visit 1 to 3 | 17.59 (32.90) | 22.10 (32.78) | 13.01 (32.49) | **0.0205** |
| SATMED - Convenience of use | Visit 1 to 2 | 3.98 (25.82) | 4.22 (26.06) | 3.74 (25.66) | 0.8724 |
|  | Visit 1 to 3 | 6.41 (27.60) | 7.80 (26.39) | 5.00 (28.79) | 0.3959 |
| SATMED - Impact on activities of daily living | Visit 1 to 2 | 9.08 (28.93) | 12.73 (27.61) | 5.46 (29.84) | **0.0324** |
|  | Visit 1 to 3 | 15.90 (35.62) | 17.73 (37.41) | 14.05 (33.75) | 0.3871 |
| SATMED - Medical care | Visit 1 to 2 | 3.65 (20.76) | 3.93 (20.58) | 3.36 (21.00) | 0.8158 |
|  | Visit 1 to 3 | 2.94 (22.54) | 1.95 (21.77) | 3.93 (23.33) | 0.4630 |
| SATMED - General satisfaction | Visit 1 to 2 | 10.19 (32.35) | 15.39 (32.39) | 4.98 (31.56) | **0.0061** |
|  | Visit 1 to 3 | 12.80 (34.28) | 16.43 (35.61) | 9.11 (32.60) | 0.0741 |
| (1) Chi-Square or ANOVA test, as per data type | | | | | |

**Table S4**. Response and remission rates at 12 weeks calculated according to the HDRS-17 scale (single-blind). We report the results for the full study population, as well as for subjects with baseline HDRS-17 score ≥ 19 for comparative purposes with previous RCT studies. Slightly lowering this baseline HDRS-17 score cutoff to ≥ 17 produces equivalent results.

| **Response and remission rates** | | | | | |
| --- | --- | --- | --- | --- | --- |
| **End point** |  | **Total** | **Study Group** | **Control Group** | **p-value (1)** |
| **Full study population (n=280)** | | | | | |
| HDRS-17 response at 12 weeks  (50% reduction from baseline) | | 120 (42.9%) | 64 (45.4%) | 56 (40.3%) | 0.3884 |
|  |  | **OR** = 1.23 (95%CI: 0.77 – 1.98) | | |  |
| HDRS-17 remission at 12 weeks  (score ≤ 7) | | 94 (33.6%) | 48 (34.0%) | 46 (33.1%) | 0.8665 |
|  |  | **OR =** 1.04 (95%CI: 0.64 – 1.71) | | |  |
| **Patients with baseline HDRS-17 ≥19 (n=150)** | |  | | |  |
| HDRS-17 response at 12 weeks  (50% reduction from baseline) | | 61 (40.7%) | 39 (49.4%) | 22 (31.0%) | **0.0221** |
|  |  | **OR** = 2.17 (95%CI: 1.11- 4.24) | | |  |
| HDRS-17 remission at 12 weeks  (score ≤ 7) | | 36 (24.0%) | 22 (27.8%) | 14 (19.7%) | 0.2444 |
|  |  | **OR** = 1.57 (95%CI: 0.73 – 3.37) | | |  |
| (1) Chi-Square test | | | | | |

**Table S5.** Type of antidepressant medications used

| **Medications used - ITT set** | | | | | |
| --- | --- | --- | --- | --- | --- |
| **Medication class** |  | **Total** | **Study Group** | **Control Group** | **p-value (1)** |
| SSRIs | N | 310 (100.0%) | 154 (100.0%) | 156 (100.0%) | 0.7412 |
|  | Yes | 138 (44.5%) | 70 (45.5%) | 68 (43.6%) |  |
|  | No | 172 (55.5%) | 84 (54.5%) | 88 (56.4%) |  |
|  | N missing | 6 | 1 | 5 |  |
| SNRIs | N | 310 (100.0%) | 154 (100.0%) | 156 (100.0%) | 0.5622 |
|  | Yes | 142 (45.8%) | 68 (44.2%) | 74 (47.4%) |  |
|  | No | 168 (54.2%) | 86 (55.8%) | 82 (52.6%) |  |
|  | N missing | 6 | 1 | 5 |  |
| TCAs | N | 310 (100.0%) | 154 (100.0%) | 156 (100.0%) | 0.6569 |
|  | Yes | 26 (8.4%) | 14 (9.1%) | 12 (7.7%) |  |
|  | No | 284 (91.6%) | 140 (90.9%) | 144 (92.3%) |  |
|  | N missing | 6 | 1 | 5 |  |
| NRIs | N | 310 (100.0%) | 154 (100.0%) | 156 (100.0%) | 0.4422 |
|  | Yes | 15 (4.8%) | 6 (3.9%) | 9 (5.8%) |  |
|  | No | 295 (95.2%) | 148 (96.1%) | 147 (94.2%) |  |
|  | N missing | 6 | 1 | 5 |  |
| Other serotonergic | N | 310 (100.0%) | 154 (100.0%) | 156 (100.0%) | 0.5443 |
|  | Yes | 73 (23.5%) | 34 (22.1%) | 39 (25.0%) |  |
|  | No | 237 (76.5%) | 120 (77.9%) | 117 (75.0%) |  |
|  | N missing | 6 | 1 | 5 |  |
| MAOIs | N | 310 (100.0%) | 154 (100.0%) | 156 (100.0%) | 0.3197 |
|  | Yes | 1 (0.3%) |  | 1 (0.6%) |  |
|  | No | 309 (99.7%) | 154 (100.0%) | 155 (99.4%) |  |
|  | N missing | 6 | 1 | 5 |  |
| (1) Chi-Square test | | | | | |

**Table S6**. Effect of the number of previously failed treatments for the current episode

| **Effect of the number of previously failed treatments for the current episode** | | | | | | |
| --- | --- | --- | --- | --- | --- | --- |
| **Treatments previously failed 🡺** | | **0** | **1** | **2** | **3** | **4 or more** |
| **Study group** | N (subjects) | 21 | 27 | 32 | 31 | 30 |
|  | HAM-D change V1 to V3 | -7.67 (8.68) | -9.19 (7.28) | -8.88 (7.92) | -8.55 (7.09) | -5.87 (7.86) |
| **Control group** | N (subjects) | 23 | 28 | 27 | 28 | 33 |
|  | HAM-D change V1 to V3 | -7.57 (6.96) | -5.79 (6.95) | -5.93 (7.69) | -5.86 (7.82) | -7.24 (6.51) |
| - Difference of averages | | 0.10 | 3.40 | 2.95 | 2.69 | -1.38 |
| - Cohen’s *d* | | **0.01** | **0.48** | **0.38** | **0.36** | **-0.19** |

**Table S7**. Efficacy results, subgroup

| **Efficacy results, Subgroups** | | | | | | |
| --- | --- | --- | --- | --- | --- | --- |
| **End point** |  | | **Total** | **Study Group** | **Control Group** | **p-value (1)** |
| **Patients having received 1 to 3 failed treatment for the current episode (n=173)** | | | | | | |
| PGI-I response at 12 weeks  (Telephone contact 3) | | | 71 (41.3%) | 44 (51.8%) | 27 (31.0%) | **0.0058** |
|  |  |  | **OR** = 2.39 (95%CI: 1.28 – 4.44) | | |  |
| HDRS-17 change | | Visit 1 to 2 | -6.17 (6.41) | -7.22 (6.14) | -5.02 (6.55) | **0.0237** |
|  |  | Visit 1 to 3 | -7.42 (7.52) | -8.86 (7.37) | -5.86 (7.40) | **0.0083** |
| (1) Chi-Square test or ANOVA according to data type | | | | | | |

**Table S8**. Tolerability results, subgroup

| **Efficacy results, ITT population (I)** | | | | | |
| --- | --- | --- | --- | --- | --- |
| **End point** |  | **Total** | **Study Group** | **Control Group** | **p-value (1)** |
| **Patients with FIBSER – Burden of side effects > 0 at baseline (Visit 1)** | | | | | |
| FIBSER - Burden  (Visit 1) | Subjects with score < 3 | 60 (33.9%) | 34 (35.1%) | 26 (32.5%) | 0.7212 |
|  | Average (SD) | 3.03 (1.22) | 3.09 (1.33) | 2.95 (1.07) | 0.4293 |
| FIBSER – Burden, subjects with score < 3 at 6 weeks (Visit 2) | | 100 (59.5%) | 64 (66.7%) | 36 (50.0%) | **0.0294** |
|  |  | **OR** = 2.00 (95%CI: 1.07 – 3.75) | | |  |
| FIBSER – Burden, subjects with score < 3 at 12 weeks (Visit 3) | | 100 (61.0%) | 63 (68.5%) | 37 (51.4%) | **0.0260** |
|  |  | **OR** = 2.06 (95%CI: 1.09 – 3.89) | | |  |
| (1) Chi-Square test or ANOVA according to data type | | | | | |
