# Supplementary material for: Efficacy of prospective pharmacogenetic testing in the treatment of major depressive disorder: results of a randomized, double-blind clinical trial
Source: BMC Psychiatry. 2017 Jul 14;17:250. doi: 10.1186/s12888-017-1412-1 (PMC5513031; doi:10.1186/s12888-017-1412-1)
Supplement: Supplementary file 2 — List of Ethic Committees that approved the AB-GEN trial. Full names of the all ethics committees that approved the trial at each participating hospital. (DOCX 13 kb) [file 12888_2017_1412_MOESM2_ESM.docx]

**List of Ethic Committees that approved the AB-GEN trial**

| **Institutional Review Board** | **Participating Center** |
| --- | --- |
| Institutional Review Board (IRB) of Hospital Clínic de Barcelona (Catalonia, Spain): *Centralized Reference IRB for the study* | Hospital Clínic, Barcelona |
| IRB of Hospital Universitari de Bellvitge (Catalonia, Spain) | Hospital Universitari de Bellvitge, Barcelona |
| IRB of Parc de Salut Mar (Catalonia, Spain) | Hospital del Mar, Barcelona |
| IRB of Fundació de Gestió Sanitaria Hospital de la Santa Creu i Sant Pau (Catalonia, Spain) | Hospital de la Santa Creu i Sant Pau, Barcelona |
| IRB of Corporació Sanitaria Parc Taulí (Catalonia, Spain) | Corporació Sanitària Parc Taulí, Sabadell |
| IRB of Consorci Sanitari de Terrassa (Catalonia, Spain) | Hospital Universitari Mútua Terrassa, Terrassa |
| IRB of Consorci Sanitari del Maresme (Catalonia, Spain) | Hospital de Mataró |
| IRB of Hospital Universitari Sant Joan de Reus (Catalonia, Spain) | Institut Pere Mata, Reus |
| IRB of Área 4 - Hospital Universitario Ramón y Cajal (Madrid, Spain) | Hospital Universitario Ramón y Cajal, Madrid |
| IRB of Área 11 - Hospital 12 de Octubre (Madrid, Spain) | Hospital Universitario 12 de Octubre, Madrid |
| IRB of Área 1 - Hospital General Universitario Gregorio Marañón (Madrid, Spain) | Hospital Universitario del Sureste, Madrid  Hospital Universitario Infanta Leonor, Madrid |
| IRB of Fundación Jiménez Díaz - UTE (Madrid, Spain) | Hospital Universitario Fundación Jiménez Díaz, Madrid |
| Comité Ético de Investigación Clínica de Galicia (Galicia, Spain) | Hospital Álvaro Cunqueiro, Vigo |
| Comité Coordinador de Ética de la Investigación Biomédica de Andalucía (Andalucia, Spain) | Hospital General de Jerez de la Frontera, Cádiz  Hospital Regional Universitario de Málaga  Complejo Hospitalario Universitario de Granada |
| Comité Ético de Investigación Clínica de Asturias (Asturias, Spain) | Hospital Universitario Central de Asturias, Oviedo |
